# The French Law of Lunacy

**Published:** 1850-07-01

**Authors:** 


### THE FRENCH LAW OF LUNACY.

### § I.

### OF LUNATIC ASYLUMS.

Article 1.—Every department is directed to have a public establishment, specially designed for the reception and treatment of lunatics, or to make arrangements for that object with a public or private establishment, either in that department or in some other.

All arrangements made with public or private establishments, are subject to the approval of the Minister of the Interior.

*Article* 2.—The public establishments devoted to lunatics are immediately under the control of the public authorities.

*Article* 3.—Private establishments for the insane are also under the surveillance of the public authorities.

*Article* 4.—The Prefect, and those individuals specially nominated by him for that purpose, or by the minister of the interior, the president of the court, the king's advocate, the justice of the peace, the mayor of the division, are directed to visit the establishments, both private and public, dedicated to the service of the insane.

They will receive the petitions of those confined there, and will, respecting their interests, procure all the information requisite to give a precise knowledge of their condition.

The private establishments are to be inspected at uncertain periods, once at least every three months, by the king's advocate for the hundred. The public establishments are in like manner to be inspected once at least every half-year.

*Article* 5.—No person shall be permitted to direct or form a private asylum for lunatics without the sanction of the government.

Private establishments intended for the treatment of other diseases, cannot admit patients labouring under lunacy, unless they be received into a detached house.

These establishments for that purpose require to receive the special sanction of the state, and will be subjected in every respect, so far as the lunatics are concerned, to all the regulations enforced by law.

*Article* 6.—The regulations of the administrative government determine the conditions under which the authority announced in the preceding article will be granted, the cases in which they are to be withdrawn, and the obligations by which the authorized establishment are to be bound.

*Article* 7.—The internal arrangements of houses for insane, either in whole or in part, shall, in so far as that public branch is interested, be submitted to the sanction of the minister of the interior.

### § II.

### ON THE RECEPTION OF PATIENTS INTO THE LUNATIC ASYLUMS.

CLAUSE FIRST.—*Of Voluntary Admissions.*

*Article* 8.—The heads or responsible governors of public establishments, and the directors of private asylums for the insane, are not to receive any one labouring under lunacy, unless there be transmitted with the party—

1. A claim for admission, with the name, profession, age, and abode, both of the person who makes it, as well as of the individual for whom it is intended, with the precise degree of relationship subsisting between the parties, or failing that, the mutual relations between them.

The petition is to be written and signed by the individual who draws it up, and if he be unable to write, it will be received by the mayor, or the commissary of police, who will ratify it.

The heads, governors, or directors, on their own responsibility, will have to satisfy themselves of the identity of the person who has drawn up the petition, in case it has not been received by the mayor, or the commissary of police.

If the claim for admission is drawn up by the guardian of the interdicted person, he will be required to present an extract of the decree of interdiction, in confirmation thereof.

2. A certificate from a physician, attesting to the mental condition of the person whose admission is proposed, pointing out the peculiarities of the disorder, and the expediency of treating the individual described in a lunatic asylum, and confining him there.

This certificate is not to be acknowledged if it has been written more than fifteen days before it is sent to the governor or director of the lunatic asylum; if it is given by a physician attached to the institution, or if the attesting physician is a relation or connexion, to the second degree inclusive, of the governor or proprietor of the establishment, or of the individual who is to place the patient in the asylum.

In cases of an urgent nature, the heads of public establishments may dispense with a medical certificate.

3. The passport, or some other document of the personal identity of the proposed patient.

In the bulletin of admission, mention will be made of all the documents produced, which will be transmitted in the course of twenty-four hours, with a certificate of the physician of the institution, and the copy of that above-mentioned, to the prefect of the police at Paris, to the prefect, or sub-prefect in the principal parishes of the department or hundred, and to the mayors in the other parishes. The sub-prefect or mayor shall immediately transmit it to the prefect.

*Article* 9.—If the order is for a person to be confined in a private establishment, the prefect, within three days from the receipt of the bulletin, will direct one or more professional man to visit the person described in the bulletin, so as to ascertain the state of his mind, and furnish an immediate report. He may associate any other person he thinks fit with him or them.

*Article* 10.—During this time the prefect will notify officially the names, profession, and abode, both of the proposed patient, and also of the person who makes the petition for the admission, and the grounds for confinement:—1st. To the king's advocate for the hundred of the dwelling-house of the lunatic. 2nd. To the king's advocate of the hundred of the locality of the asylum. These instructions apply both to public and private institutions for the insane.

*Article* 11.—Fifteen days after the reception of a patient, either in a public or private asylum, a new certificate of the physician of the institution is to be addressed to the prefect, in conformity with the last paragraph of Article 8. This certificate will corroborate, or, if it be necessary, rectify the remarks delivered in the first, stating the more or less frequent recurrences of the paroxysms, or of the acts of dementia.

*Article* 12.—There is to be kept in every institution, a register, endorsed and signed by the mayor, in which are to be immediately inserted the names, professions, ages, and abodes of the individuals confined in the lunatic asylums; the statement of the decree of interdiction, if it has been delivered, with the name of the guardian; the dates of their admission; the names, profession, and abode of the individual, whether relation or not, who has given in the petition. The following also is to be inscribed in the register:—1st. The certificate of the physician in connexion with the claim for admission. 2. Those certificates which, in conformity with Articles 8 and 11, the physician of the institution is required to transmit to the government.

In the register, at least every month, the physician is directed to report any changes that may have taken place in the mental condition of every patient. The dismissals and deaths are likewise to be recorded in the register.

This register is to be placed for examination before those parties who have the authority and privilege to inspect the asylum, when they visit the place for that purpose. After having concluded the inspection, they will place on the register their visa, with their signature, and observations, if there be any necessary.

*Article* 13.—Every individual confined in a lunatic asylum, ceases to be detained there as soon as the physicians of the establishment have declared in the register referred to in the preceding article, that the cure has been effected.

If the individual is a minor, or under interdiction, notice of the opinion of the physicians will be given to the person to whom it ought to be sent, and to the king's advocate.

*Article* 14.—Before the physicians declare that a cure is accomplished, every person placed in a lunatic asylum is free to leave it, when his dismissal is required by any of the individuals undernoted; to wit—

1. The curator appointed according to Article 38 of the present law.

2. The husband or wife.

3. If there are neither husband nor wife, the elder relative.

4. Failing these, the junior relatives.

5. The individual who has signed the claim for admission, provided no relation is opposed to the use of that authority, without the assent of the council of the family.

6. Any person authorized to do so by the sanction of the council for the family.

If there be any opposition notified to the chief of the asylum by any having authority, that there is a difference of opinion, either amongst the elder relatives, or amongst the junior, the advocate of the family will decide.

Nevertheless, if the physician of the asylum be of opinion, that public order or the safety of individuals would be compromised by the enlargement of the lunatic, he will give notice thereof previously to the mayor, who will immediately direct a provisional *supersedeas* to the order for dismissal, on condition that it be reported to the prefect in twenty-four hours. This provisional supersedeas will lose all force in law after the expiry of a fortnight, if the prefect has not during the interval given contrary directions, in conformity with Article 21, below. The order of the mayor is to be transcribed into the Register kept in accordance with Article 12.

In case of minority or of interdiction, the guardian alone may authorize the dismissal of the patient.

*Article* 15.—Within twenty-four hours after dismissal, the governors or directors will give notice of it to the functionaries mentioned in the last paragraph of the Article 8; and will intimate to them the name and residence of the individuals who have withdrawn the patient, the state of his mind at the moment when he was enlarged, and in particular, if possible, the locality where he has gone to.

*Article* 16.—It is always in the power of the prefect to direct the immediate discharge of persons under voluntary confinement in lunatic asylums.

*Article* 17.—In every instance the person under interdict can only be restored to his guardian, and the minor to those who have legal authority for his control.

### Clause Second.—*Of Cases admitted under Public Authority.*

*Article* 18.—At Paris, the prefect of police, and in the departments, the prefects, will regulate the order for admission into a lunatic asylum, of every individual, whether interdicted or not interdicted, whose state of mind is such as to compromise public order, or the safety of individuals.

The orders of the prefects ought to give the reasons, and state the circumstances which rendered them necessary. These orders, as well as those delivered in accordance with Articles 19, 20, 21 and 23, shall be inserted in a Register similar to that directed by Article 12 above, all the forms of which are applicable to persons confined officially.

*Article* 19.—In all cases of insanity attended by imminent danger, certified by the statement of a physician, or declared through public notoriety, the commissary of police at Paris, and the mayors in the other departments, will direct every provisional measure that may be requisite, under condition that they are reported within the period of twenty-four hours, to the prefect, who will deliver his decree thereon without delay.

*Article* 20.—The heads, directors, or governors responsible for lunatic asylums, are by law bound to write to the prefects, in the first month of every half year, a Report prepared by the physician of the institution, on the condition of each individual under confinement, containing an account of the nature of the disease, and the result of the treatment.

The prefect shall deliver his opinion on each individually, and order his continuance in the asylum, or his discharge.

*Article* 21.—In respect of individuals confined voluntarily, and in those instances where the state of the mind might be such as to compromise public order, or the safety of individuals, the prefect may, in the forms specified by the second paragraph of Article 18, give a special order, to the effect that the individual be prevented leaving the asylum, without his sanction, unless it be to become the inmate of another institution.

The heads, directors, or responsible governors, are bound to act in conformity with this rule.

*Article* 22.—Intimation must be given to the king's advocates of all orders or directions delivered in virtue of the Articles 18, 19, 20, 21.

These orders shall be intimated to the mayor of the abode of the persons placed under restraint, who will notify it immediately to the families.

An account shall also be transmitted to the minister of the interior.

The different notifications prescribed by the present article shall be made in pursuance of the forms and retardations detailed in Article 10.

*Article* 23.—If, during the interval which shall elapse between the Reports directed by Article 20, the physicians declare on the Register kept in conformity with Article 12, that the discharge may be permitted, the heads, directors, and responsible governors of the asylums, shall be bound under pain of prosecution, agreeably to Article 31, below, to make an immediate report to the prefect, who will ordain without delay.

*Article* 24.—The *hospices* and hospitals are bound provisionally to receive those persons transmitted to them in virtue of the Articles 18 and 19, until quarters are found for them in the asylums specially intended for their reception, according to the terms of the First Article, or during their passage to these places.

In all the parishes where there are religious houses or hospitals, the lunatics cannot be disposed of otherwise than in these houses or hospitals. Where there are none, the mayors shall be enforced to provide lodgings for them, either in an inn, or in a dwelling hired for the purpose.

In no instance are lunatics to be transported along with criminals, or those condemned, nor lodged in a prison.

These arrangements apply to all lunatics placed by government either in a public or private asylum.

### Clause Third.—*Of the Expenses of Lunatics.*

*Article* 25.—Lunatics placed in confinement by order of the prefect, and whose families have not petitioned for their admission into a private asylum, shall be conveyed to the asylum for their own department, or to such other with which an agreement has been made.

Those lunatics whose state of mind would not affect public order, nor the personal safety of individuals, shall have free admission there, in the modes, under the circumstances, and according to the conditions which will be regulated by the general council, on the proposal of the prefect, and sanctioned by the minister.

*Article* 26.—The expense of the conveyance of persons directed by the administration of lunatic asylums, will be regulated by the prefect, on the report of the parties charged with the conveyance.

The expense for maintenance, lodging, and treatment, of persons placed in religious houses, or public lunatic asylums, will be regulated according to a tariff fixed by the prefect.

The expenses of maintenance, lodging, and treatment of persons confined by the authority of the departments in private asylums, shall be fixed by the arrangement made by the departments, agreeably to Article 1.

*Article* 27.—The expenses mentioned in the preceding Article shall be charged to the persons confined—failing whom, on those for whom they would have a claim for aliment, agreeably to the directions in Article 205 of Civil Code.

If the obligation to furnish the food be disputed, or the quota contested, a competent tribunal will issue a decree thereon, on the prosecution of the trustee appointed to act in accordance with Article 31 and 32.

The recovery of money due will be prosecuted at the instance of the administration for enrolment, and for property.

*Article* 28.—In default, and when the aforesaid sources are inadequate, it will be provided by the financial laws, at the ordinary expense of the department to which the lunatic belongs, without prejudice to the meeting of the commune where the lunatic resided, according to a basis submitted by the general council at the instance of the prefect, with the sanction of the government.

The religious houses shall have claim for an indemnity, corresponding with the number of the lunatics placed at their charge, and who shall be confined in an asylum appropriated to lunatics.

In case of differences, the council of the prefecture shall give a final decision.

CLAUSE FOURTH.—*Rules and Arrangements common to all individuals confined in Lunatic Asylums.*

*Article* 29.—Every individual confined or detained in a lunatic asylum, the guardian, if he be a minor, his tutor, any relative or friend, at any period, may, by making application before the tribunal in the immediate locality of the asylum, after the proper authentications are lodged, obtain an order for the immediate discharge of the patient.

The persons who shall have demanded the confinement, and the king's advocate, may make similar applications.

In the case of interdiction, this application can only be drawn up by the guardian of the person interdicted.

The decision will be delivered simply by making application in the council-room, without giving the reasons.

The application, the decision, and the other deeds to which the reclamation will give rise, will be examined for stamp, and registered in the account.

Any requests, any petitions addressed, either to the judicial or to the administrative bodies, must not be suppressed or retained by the directors of the asylums, under the penalties mentioned under the Third Section, above.

*Article* 30.—The governors, directors, or responsible superintendents, are not permitted, under the penalties mentioned under the 120th Article of the Penal Code, to retain any individual confined in a lunatic asylum, when the discharge has been ordered by the prefect, according to the directions of the Articles 12, 20, and 23, or by the court, according to the terms of the 29th Article, except the conditions of the Articles 13 and 14 apply to the case.

*Article* 31.—The administrative commissions, or those entrusted with the superintendence of hospitals or public lunatic asylums, shall exercise, as regards persons not interdicted, but under confinement, all the provisional functions of administration. They shall select one of their body to fulfil those functions. The individual so authorized, will take the usual steps for the recovery of monies due to the person in confinement and for the discharge of the debts, will give leases which are not to exceed the limit of three years, and may even, in virtue of special power delegated by the civil tribunal, sell the property.

The proceeds from the sale, or from other sources, will be put directly into the treasury of the establishment, and, if necessary, shall be expended for the interest of the lunatic.

The caution, or bail, for the receiver will be found as security of above money, by a claim on every other kind of debt.

Nevertheless, the relations, the husband, or the wife of the person confined in the lunatic asylum, under the direction and •guidance of the administrative commission, the commission itself, and the king's advocate likewise, may have their appeal to the subsequent Articles.

*Article* 32.—On the demand of the relatives, or of the husband, or of the wife, along with that of the administrative commission, or on the official charge of the king's advocate, the civil court in the locality of the dwelling of the lunatic may, in conformity with Article 497 of the Civil Code, nominate in the council chamber a provisional curator for the good of any person not under interdiction in confinement in a lunatic asylum. The nomination will not be final until after the family shall have advised thereon, and the conclusions of the king's advocate been heard. There will be no appeal from it.

*Article* 33.—The court, at the request of the provisional curator, or by command of the king's advocate, will appoint a special mandatory to represent judicially every individual not under an interdict, and confined in a lunatic asylum, who will be engaged as judicial agent at the date of confinement, or against whom an action may be afterwards raised.

The court shall also, in a case of urgency, nominate an especial mandatory, to commence a suit, in the name of the same individuals, for moveable or fixed property. In which two cases, the provisional curator may receive the appointment of special mandatory.

*Article* 34.—The arrangements of the civil codes as to the causes which do not require tutelage, as affects incapacity, the exclusion, or the destitution of tutors, apply to all provisional curators nominated by the court.

On the petition of interested parties, or that of the king's advocate, the decree, which fixes the provisional curator, may at the same time determine a general or special mortgage on his property, so as to be responsible for the sum arranged by the aforesaid judgment.

The king's advocate ought, in the course of a fortnight, to cause the mortgage to be registered in the office for deeds: it only takes its date from the time it is inscribed.

*Article* 35.—When the provisional curator is appointed by a decree, the communications to be made to the lunatic are to be made to that curator.

Communications presented to the house may, according to circumstances, be annulled by the courts.

Nothing here interferes with the provisons of the Article 173 of the Code of Commerce.

*Article* 36.—In default of a provisional curator, the president, on the application of the most attentive party, will authorize a notary to represent the interests of individuals not under interdiction, confined in lunatic asylums, as to inventories, accounts, shares, and disbursements, which may affect them.

*Article* 37.—The authority conferred through the sanction of the preceding articles ceases entirely on the person being removed from the asylum.

The powers sanctioned by the court, in virtue of the 32nd Article, cease entirely after the expiration of three years: they may, however, be renewed.

This arrangement does not, however, apply to provisional curators appointed for individuals maintained by the authorities in private establishments.

*Article* 38.—On the petition of a person who has an interest in the matter, one of his relations, of a husband or of a wife, of a friend, or at the instance of the king's advocate, the court may, in a chamber of consultation, nominate by a decree, against which there shall be no appeal, in addition to the provisional governor, a curator to the person of each individual confined in a lunatic asylum, but not under interdiction, whose duty will be to see—1st, that his income is employed to soothe his unhappy fate, and to promote his cure; 2nd, that the aforesaid individual is restored to the free exercise of his rights, as soon as the condition of his circumstances will permit.

This curator, or guardian, cannot be selected out of the presumptive heirs of the person in confinement.

*Article* 39.—The deeds of a person placed in a lunatic asylum, during the period of his confinement, though there has been no interdiction certified or raised, may be brought in evidence for dementia, agreeably to Article 1304 of the Civil Code.

The ten years of action for nullifying the deeds shall run, as refer to the lunatic who shall have subscribed these acts, commencing at the date at which intimation has been given him, or of the knowledge he has received of them after his final discharge from the lunatic asylum.

And in regard to his heirs, from the date of the intimation which has been sent, or of the knowledge they may have acquired thereof, since the death of the individual.

The period of the beginning of the ten years against the one individual is also that of the ten years against the heirs.

*Article* 40.—The public minister will be heard on all matters affecting the interests of individuals confined in lunatic asylums, even though they are not under interdiction.

### § III.

### GENERAL ARRANGEMENTS.

Disobedience to, or infraction of, the measures sanctioned by Articles 5, 8, 11, 12, of the second paragraph of Article 13, of the Articles 15, 17, 20, 21, and of the last paragraph of Article 29

of the existing law, and to the rules rendered under the authority of the 6th Article, on the part of the heads, directors, or responsible governors of public or private lunatic asylums, and by the physicians officiating therein, shall be punished by imprisonment from five days to a year, and a fine from fifty francs to three thousand francs, or by one or other of those amercements.

The Article 463 of the Penal Code may be enforced.

The present law, discussed, debated, and adopted by the Chamber of Peers and the Chamber of Deputies, and sanctioned by us this day, is henceforth the law of the realm.

Given at the Palace of Neuilly, the 30th day of the month of June, 1838.*

(*Signed*) LOUIS PHILIPPE.

---

### MR. DYCE SOMBRE ON THE ENGLISH LAW OF LUNACY.

* This law has, we believe, undergone some trifling amendments and additions, but in its main provisions it remains as above detailed.—ED.

